# Mitochondrial DNA Is a Vital Driving Force in Ischemia-Reperfusion Injury in Cardiovascular Diseases

**DOI:** 10.1155/2022/6235747

**Published:** 2022-05-17

**Authors:** Hui Liu, Xin Liu, Jingxin Zhou, Tao Li

**Affiliations:** ^1^Department of Anesthesiology, Laboratory of Mitochondria and Metabolism, National-Local Joint Engineering Research Centre of Translational Medicine of Anesthesiology, Chengdu 610041, China; ^2^Department of Anesthesiology, West China Second University Hospital, Sichuan University, Key Laboratory of Birth Defects and Related Diseases of Women and Children, Sichuan University, Ministry of Education, Chengdu 610041, China

## Abstract

According to the latest Global Burden of Disease Study, cardiovascular disease (CVD) is the leading cause of death, and ischemic heart disease and stroke are the cause of death in approximately half of CVD patients. In CVD, mitochondrial dysfunction following ischemia-reperfusion (I/R) injury results in heart failure. The proper functioning of oxidative phosphorylation (OXPHOS) and the mitochondrial life cycle in cardiac mitochondria are closely related to mitochondrial DNA (mtDNA). Following myocardial I/R injury, mitochondria activate multiple repair and clearance mechanisms to repair damaged mtDNA. When these repair mechanisms are insufficient to restore the structure and function of mtDNA, irreversible mtDNA damage occurs, leading to mtDNA mutations. Since mtDNA mutations aggravate OXPHOS dysfunction and affect mitophagy, mtDNA mutation accumulation leads to leakage of mtDNA and proteins outside the mitochondria, inducing an innate immune response, aggravating cardiovascular injury, and leading to the need for external interventions to stop or slow the disease course. On the other hand, mtDNA released into the circulation after cardiac injury can serve as a biomarker for CVD diagnosis and prognosis. This article reviews the pathogenic basis and related research findings of mtDNA oxidative damage and mtDNA leak-triggered innate immune response associated with I/R injury in CVD and summarizes therapeutic options that target mtDNA.

## 1. Introduction

Cardiovascular disease (CVD), which includes ischemic heart disease (IHD), rheumatic heart disease, congenital heart disease (CHD), stroke, heart failure (HF), peripheral arterial disease, and several other heart and vascular diseases, is the leading cause of death worldwide and a major cause of reduced quality of life [1, 2]. According to the latest Global Burden of Disease Study, there were an estimated 5.23 billion patients with CVD in 2019, and CVD has caused 18.6 million deaths [1]. IHD is the leading cause of death in patients with CVD. IHD is caused by coronary atherosclerosis, which involves blockage of the blood vessel lumen, resulting in myocardial ischemia and hypoxia [3]. During recanalization, normal perfusion of the ischemic myocardium is restored, but tissue damage gradually increases due to hypoxia and intracellular acidification. In addition, the generation of endogenous damage-associated molecular patterns (DAMPs) following myocardial ischemia-reperfusion (I/R) injury leads to activation of the innate immune response, exacerbating myocardial I/R injury and cell death [4].

Cardiomyocytes are highly dependent on mitochondria for energy, as the energy required for metabolism, excitatory transmission, and contraction is provided by mitochondria. Mitochondrial dysfunction leads to the occurrence and development of CVD, and oxidative stress, reduced mitochondrial biosynthesis, and mtDNA mutation or damage are the main factors leading to mitochondrial dysfunction [5]. When mitochondrial dysfunction reaches a certain level, the mitochondrial membrane and cell membrane are damaged, content leaks out of the cell, and the leaked material is recognized by pattern recognition receptors (PRRs) as endogenous DAMPs, inducing an innate immune response [6]. Since the full human mtDNA genome was sequenced in 1981, research on the association of mtDNA with CVD has received widespread attention [7]. In recent years, numerous studies have shown that during mitochondrial oxidative phosphorylation (OXPHOS) and the mitochondrial life cycle (MLC), when mtDNA undergoes oxidative damage, mtDNA mutations, mtDNA copy number (mtDNA-CN) reduction, and mtDNA leakage as a DAMP-triggered defense response are closely associated with the development of CVD [8].

Recent studies have suggested that mtDNA is an important driver of I/R injury in CVD and can be used as a marker for the early diagnosis and prognosis of CVD [9–12]. This article focuses on the pathogenesis of mtDNA oxidative damage or mutation and related studies and innate immune responses triggered by mtDNA leakage associated with myocardial I/R injury.

## 2. Structure and Characteristics of mtDNA

mtDNA is independent of nuclear chromosomes and has self-replicating, transcriptional, and coding functions [13]. mtDNA is a double-stranded DNA molecule in the form of a loop, with the outer loop being the heavy strand (H-strand) and the inner loop being the light strand (L-strand). Human mtDNA contains a total of 16569 base pairs, including noncoding and coding regions. The noncoding region, also known as the displacement loop (D-loop), is the control region of the mitochondrial genome. The coding region encodes 37 genes, including 2 rRNA genes, 22 tRNA, genes and 13 polypeptide genes. rRNA genes and tRNA genes are involved in the transport and synthesis of 13 polypeptides encoded by mitochondria, all of which comprise the respiratory chain complex and are involved in mitochondrial OXPHOS [14].

During I/R, mtDNA in cardiac cells is susceptible to oxidative damage for the following reasons: (1) mtDNA is closely related to the electron transport system: it exists within the mitochondrial matrix or is attached to the inner mitochondrial membrane, is easily exposed to high levels of reactive oxygen species (ROS) and other free radicals generated by the respiratory chain, and is susceptible to oxidative damage [15]. (2) mtDNA has a small molecular weight: individual mitochondrial genes are located on a single, circular DNA molecule with no nuclear membrane envelope or histones, making mtDNA susceptible to oxidative damage by ROS even if it binds to nucleoid proteins such as TFAM to form a nucleoid structure [16, 17]. (3) mtDNA has a compact gene sequence arrangement: except for the D-loop region, which is associated with mtDNA replication and transcription, all regions are devoid of intron sequences [7]. Some bases are part of two different genes; i.e., a base that serves as the end of one gene may also serve as the beginning of the next gene [18]; thus, even small changes in the mtDNA sequence due to ROS oxidation may result in changes in structurally important genes within the mtDNA genome. (4) Excessive production of ROS mediates mtDNA damage and mutations: mtDNA mutations alter tRNA structure, which affects protein synthesis and leads to defective OXPHOS, further increasing ROS production and exacerbating mtDNA mutations, forming a vicious cycle [19].

## 3. mtDNA Damage and Repair and Mitochondrial Processes

The heart is the organ with the highest energy demand in the body, with mitochondria accounting for 30% of the cardiomyocyte volume and producing approximately 95% of the adenosine triphosphate (ATP) [20–22]. To meet this high energy demand, cardiac mitochondria are equipped with two complex systems that work in concert: the OXPHOS system and the MLC system (including mitochondrial fission and fusion, mitophagy, and mitochondrial biogenesis). The proper functioning of these two systems is inextricably linked to the degree of damage and repair capacity of mtDNA in cardiac cells [23].

OXPHOS involves the electron transport chain (ETC) and an ATP synthase (ATPase) [24]. The most common step in the OXPHOS process that leads to mtDNA damage is oxidation by ROS. When the ETC is dysfunctional or the body's antioxidant defense system is weakened, ROS are generated in large quantities, triggering a cellular oxidative stress response. When I/R damage occurs in the heart, free radicals can directly damage proteins through oxidation or oxidize lipids to form lipid peroxides to exert cytotoxic effects, leading to cardiomyocyte mtDNA damage [25].

Oxidative damage to mtDNA is mainly repaired during MLC, including by a variety of enzymes required for the synthesis and activation of repair pathways.

### 3.1. Mitochondrial Dynamics

Mitochondrial dynamics mainly refers to mitochondrial fission and fusion, which play a key role in maintaining cellular homeostasis. Mitochondrial fusion mainly repairs damaged mitochondria by interchanging matrix proteins and mtDNA in the mitochondrial matrix. Mitochondrial fission mainly results in the redistribution of mitochondrial substances, cytochrome c (Cyt c) release, apoptosis, and mitochondrial degradation. The molecules responsible for these processes include fusion proteins (Mitofusin 1 (Mfn1), Mitofusin 2 (Mfn2), and optic atrophy 1 (OPA1)), fission mediators (dynamin-related protein 1 (Drp1)), and mitochondrial receptor protein mitochondrial fission 1 (Fis1), mitochondrial fission factor (MFF), and mitochondrial dynamics proteins of 49 and 51 kDa (Mid49 and Mid51, respectively), which interact to ensure mitochondrial quality control [26]. Defects in these proteins can lead to loss of mtDNA integrity, impairment of mitochondrial function, severe alterations in mitochondrial morphology, and ultimately cell death [27]. For example, studies have shown that conditional ablation of cardiac-specific MFN2 (*α*-MHC-Cre) leads to mitochondrial pleomorphism and mild enlargement, as well as moderate hypertrophy of the left ventricle and mild deterioration of systolic function [28, 29]. In myocardial ischemia, excessive mitochondrial fission and reduced mitochondrial fusion cause mitochondrial dysfunction [30]. Activated adenosine 5′-monophosphate- (AMP-) activated protein kinase (AMPK) can inhibit Drp1 phosphorylation, attenuate mitochondrial fission, and protect against myocardial ischemia [30]. A recent study has found that secreted frizzled protein 5 (Sfrp5), a wingless-type (Wnt) signaling antagonist and an anti-inflammatory adipokine, reduces myocardial ischemia by activating AMPK phosphorylation, increasing the expression of mitochondrial fusion proteins (Mfn1 and Mfn2), and inhibiting mitochondrial fission proteins (p-Drp1^Ser616^/Mid49/MFF/Fis). The upregulation of Sfrp5 by sirtuin 1- (SIRT1-) Liver kinase B1 (LKB1)-AMPK signaling pathway could restore mitochondrial function and attenuate myocardial ischemia-reperfusion injury [31]. Interestingly, Sfrp5 is also a potential therapeutic target as its serum level is associated with early improvement of cardiac function in acute ST-segment elevation myocardial infarction (STEMI) patients [32].

### 3.2. Mitophagy

Excessive cytoplasmic components are “confiscated” by autophagic vesicles, which subsequently fuse with lysosomes and are degraded by deoxyribonuclease (DNase) in lysosomes to eliminate damaged mtDNA [33, 34]. Incomplete digestion of mtDNA leads to infiltration of thoracic transverse aortic constriction-operated cardiac inflammatory cells and production of proinflammatory cytokines and leads to cardiac insufficiency and HF [35]. Mitophagy can limit excessive inflammatory responses by regulating key immunomodulators such as nuclear factor-*κ*B (NF-kB) and STING [36, 37]. Under pathological conditions, when the accumulation of dysfunctional mitochondria and damaged mtDNA in the cytoplasm exceeds the capacity of the cell to degrade these components by mitophagy, the negative regulatory function of mitophagy is abolished, resulting in a significantly elevated inflammatory response [38, 39].

### 3.3. Mitochondrial Biogenesis

The entire processes of mitochondrial production and proliferation are regulated by both mtDNA and nuclear DNA (nDNA). Peroxisome proliferator-activated receptor *γ* coactivator 1*α* (PGC-1*α*) is the most important signaling molecule in the regulation of mitochondrial production [40]. PGC-1*α* regulates mitochondrial production mainly by cotranscribing mitochondrial constituent proteins and mitochondrial transcription factor A (mtTFAM) with nuclear respiratory factors 1 and 2 (NRF1 and NRF2). mtTFAM in turn controls mtDNA replication and transcription [41]. During myocardial I/R injury, multiple molecules exert protective effects on the myocardium through activation and promotion of PGC-1*α*-mediated mitochondrial biogenesis [42–44]. For example, the histone deacetylase (HDAC) inhibitor suberoylanilide hydroxamic acid (SAHA) was shown to induce cardiomyocyte mitophagy and attenuate I/R injury during reperfusion. In vivo, I/R was found to decrease mtDNA content in the marginal zone of the heart in >50% of mice, whereas pretreatment with SAHA alone and reperfusion restored mtDNA content and mitochondrial mass to normal levels by increasing the expression of the PGC-1*α* gene [42]. Studies have shown that depletion of mtDNA due to impaired mitochondrial biogenesis is a common feature of myocardial hypertrophy and end-stage ischemic HF [45].

## 4. mtDNA Mutations and CVD

mtDNA mutations may be due to unrepaired mtDNA damage [46] or lesions resulting from miscoding due to spontaneous errors in DNA replication [47]. There are very few noncoding genes between adjacent mtDNA genes, leading the mutation rate of mtDNA to be 10-20 times higher than that of nDNA [48]. The number of mtDNA copies in cardiomyocytes ranges from 4,000 to 34,000 [49], and deleterious mutant genes often affect only part of the mtDNA; therefore, both mutant and wild-type (WT) mtDNA can exist within the same mitochondria or between different mitochondria, which is called heterogeneity [50]. The greater the heterogeneity (the proportion of mutations) is, the more likely the heart is to have abnormalities. During ischemia and recurrent hypoxia, the production of large amounts of oxygen radicals by cardiomyocytes can cause irreversible damage to mtDNA, leading to mtDNA mutations [51]. If the proportion of mtDNA mutations in cardiomyocytes exceeds a certain threshold (60-70%), cellular homeostatic mechanisms are disrupted, resulting in mitochondrial dysfunction, cell atrophy, and death, leading to the development of CVD [52].

Recently, researchers deeply sequenced the complete mtDNA genome of 356 cardiac patients and identified 11 mtDNA heterogeneous “hotspots” covering major genes, including NADH dehydrogenase (ND) subunits 1, 4, 5, and 6; cytochrome c oxidase I (CoI); 16S rRNA; and D-loop. Mutations in the ND and CoI genes directly affect ATP production [53]. In addition, reports have shown that mtDNA mutations also affect mitochondrial autophagy [54]. mtDNA mutations selectively disrupt the differentiation-induced activation of mitophagy in adult cardiac precursor cells (CPCs), and loss of Bcl-2 interacting protein 3 like (BNIP3L) and FUN14 domain containing 1- (FUNDC-1-) mediated mitophagy during differentiation in CPCs leads to sustained mitochondrial fission and an increased risk of myocardial infarction (MI) [55]. The relevance of mutations in the D-loop region to CVD susceptibility was reported in a recent study that showed a high proportion of point heterogeneity in the D-loop region in controls with MI, implying that mtDNA heterogeneity may also have a protective effect [56].

### 4.1. Common Types of mtDNA Mutations

#### 4.1.1. mtDNA Base Deletion

Deletion of mtDNA bases disrupts the normal function of the respiratory chain and reduces proton flow, thereby reducing mitochondrial membrane potential and inhibiting mitochondrial ATP synthesis.

#### 4.1.2. Point Mutations in tRNA Genes Associated with Protein Synthesis

Mutations in tRNA are expected to disrupt base pairing at the affected site, potentially altering the secondary structure of this tRNA and leading to its more rapid degradation and subsequent reduction in mitochondrial protein levels [57].

#### 4.1.3. Mutations in Structural Genes Encoding Subunits of the Mitochondrial Respiratory Complex

Mutations in this region reduce ATP synthesis and increase ROS production.

#### 4.1.4. Mutations in the D-Loop Region

This region contains three hypervariable regions, the initiation site of mtDNA replication and the transcriptional promoter, which have regulatory effects on mtDNA replication and transcription [58, 59]. Mutations in this region would lead to a reduction in mtDNA-CN.

#### 4.1.5. A Decrease in the mtDNA-CN

The mtDNA-CN represents the number of mitochondria per cell and the number of mitochondrial genomes per mitochondrion and is an indirect biomarker of mitochondrial function. Reduced mtDNA-CN must cause abnormal mitochondrial function, and some investigators have used this feature to improve risk classification for CVD [60].

### 4.2. mtDNA Mutations and IHD

IHD, also known as coronary artery disease, is characterized by atherosclerosis, which leads to the narrowing or blockage of the lumen of blood vessels, resulting in angina and MI. The large amount of oxygen free radicals produced by cardiomyocytes during ischemia and repeated hypoxia will cause irreversible damage to mtDNA, leading to mtDNA mutations [51]. mtDNA mutations lead to the exacerbation of OXPHOS, generating more ROS during IR, leading to impaired ATP production and oxidative stress, which may further accelerate the development of or susceptibility to atherosclerosis and myocardial ischemic injury that forms a vicious cycle [61]. However, it is unclear whether mtDNA mutations are a cause or an effect of IHDs [62].

mtDNA base deletions are frequently found in patients with IHD. Researchers found that the number of mtDNA deletions in patients with IHD far exceeded that in controls [63]. The more severe the degree of cardiac ischemia is, the higher the rate of deletion mutations in cardiomyocytes [63], especially deletion of the 4977 bp fragment of mtDNA, the rate of which is 7-220 times higher in patients with cardiac ischemia than in normal individuals [64]. Recent studies have shown that tRNA genes are also hotspots for pathogenic mutations associated with IHD, such as the A5592G, C3256T, G15927A, and T15062C mutations [65]. Mutations in G12315A affect protein synthesis and thus lead to enzymatic defects in the mitochondrial respiratory chain, which may play a role in MI [66]. In addition, a meta-analysis used the mtDNA-CN to predict the risk and prognosis of CVD and showed that the lower the mtDNA-CN is, the higher the risk of developing IHD [67].

### 4.3. mtDNA Mutations and Non-IHD

#### 4.3.1. Primary Cardiomyopathies

Primary cardiomyopathies are a group of cardiac diseases with an unknown cause, mainly myocardial lesions, combined with hypocardial function [68]. It has been found that mtDNA in cardiomyocytes in primary cardiomyopathy is often mutated and missing, and mitochondrial dysfunction occurs. Additionally, cardiomyopathy is the most common form of CVD in patients with mitochondrial disease [61, 69]. The main mtDNA mutations associated with cardiomyopathy include point mutations in tRNAs related to protein synthesis, mutations in structural genes encoding subunits of the mitochondrial respiratory complex, and deletions of mtDNA fragments. One study examined mtDNA mutations in endomyocardial biopsies from 85 patients with significant mitochondrial morphological alterations, and a total of 18 mutations, nine of which were located in tRNA (e.g., A3243G, A3260G, A4300G, and A4317G), were identified [70]. Mutations in structural genes encoding subunits of the mitochondrial respiratory complex are also common in cardiomyopathies. For instance, these mutations are found in Cytb, CoI (e.g., C6521G), CoII (e.g., A7673G), and ND6 (e.g., T14180C). The most studied mutant gene is Cytb, and several structural mutation loci encoding subunits of the mitochondrial respiratory chain complex (e.g., A15236G, C15452A, and A14927G) that result in amino acid alterations and cardiac symptoms have been identified to be associated with cardiomyopathies [71].

#### 4.3.2. Hypertensive Disease

Hypertensive disease is a chronic disease characterized by a sustained increase in arterial blood pressure [72]. Several recent studies have identified multiple mtDNA mutations associated with hypertension disease, suggesting a maternally inherited trait [73, 74]. Only most of the relevant studies have been conducted in Chinese populations [75, 76]. In hypertensive disease, common mutations occur in the D-loop region and in the tRNA gene region [77]. For example, in patients with maternally inherited essential hypertension, mutations in tRNAs T4386C and C4394T were found [75].

#### 4.3.3. HF

A variety of CVDs progress to eventually develop into HF. HF is the leading cause of morbidity and mortality worldwide, and epidemiological studies have shown that more than 50% of HF is classified as HF with preserved ejection fraction (HFpEF) [78]. Up to 40% of deaths due to CVD occur in people over 65 years of age [79]. As early as 2004, animal experiments demonstrated that mutations in mtDNA are responsible for aging and CVD, with the “mutant mice” showing all the symptoms of old age and cardiac hypertrophy at the age of 6 months [80]. The animal model of HFpEF recently reported in our laboratory also shows typical symptoms of hypertension and aging [81]. As humans get older, mutations in mtDNA accumulate. A recent study performed high-depth mtDNA sequencing of blood DNA from patients with hypertension, IHD, ischemic stroke, and healthy controls and showed that the per-individual burden of heteroplasmic single-nucleotide variants increases with age [62].

In severe acute HF, the release of mtDNA is associated with increased mortality. However, in patients with aging-associated chronic HF, mtDNA predominantly exhibits a decrease in mtDNA-CN [82, 83], mtDNA point mutations, and mtDNA point deletions [84]. Theoretically, the coupling of OXPHOS and MLC could reduce mtDNA heterogeneity and restore mtDNA-CN, but with aging, the repair capacity of mitochondria also declines, leading to the accumulation of damaged mitochondria in the cell, thus aggravating HF [85].

#### 4.3.4. CHD

CHD is the leading cause of death from birth defects [86, 87]. Early investigators reported ventricular septal defects [88] and tetralogy of Fallot [89] in association with mtDNA mutations in the form of case reports. Currently, mitochondrial defects have been observed in mouse models of dysplastic left heart syndrome [90], and LONP1 (lon peptidase 1, mitochondrial) has been reported as a candidate congenital diaphragmatic hernia-associated gene [91]. These findings have piqued the interest of researchers to explore the correlation between CHD and mtDNA mutations. A recent study found that neither mtDNA-CN variants nor mitochondrial gene mutations were likely to significantly increase the risk of CHD by examining clinical samples [92].

**Figure 1:**
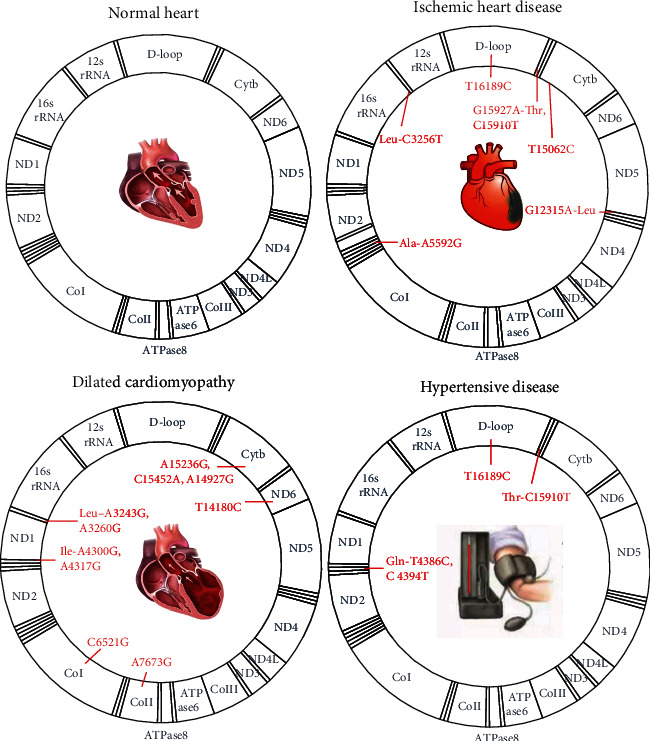
The common mitochondrial gene mutation loci in CVD include ischemic heart disease, dilated cardiomyopathy, and hypertensive disease.

After the review of the relevant papers, we have not yet found any mtDNA mutations that overlap with IHD, dilated cardiomyopathy, and hypertensive disease. However, we found three overlapping mtDNA mutations between these two diseases, including a deletion of the 7436 fragments shared by IHD [64] and dilated cardiomyopathy [93], a mutation in the C15910T tRNA between IHD [57] and hypertensive disease [94]. It was found that the T16189C mutation in the D-loop region is more common in hypertensive patients [95] and drives the progression of IHD in a Middle European population [96]. In Figure 1, we show mitochondrial gene mutation loci that are more commonly associated with CVDs, such as IHD, dilated cardiomyopathy, and hypertensive disease.

## 5. Innate Immune Response Triggered by mtDNA as a DAMP

Once the membrane potential of the mitochondrial matrix is disrupted and/or the integrity of the mitochondrial membrane is compromised, mtDNA leaks from the mitochondrial matrix into the cytoplasm [97, 98]. In addition, biological processes that disrupt the cell membrane structure, such as cell necrosis and cell scorching, result in leakage of mtDNA from the intracellular to the extracellular environment. mtDNA, whether it leaks into the cytoplasm or the extracellular space, can act as a DAMP that binds to PRRs inside and outside the cell to trigger an innate immune response, leading to myocardial hypertrophy, fibrosis, accumulation of misfolded proteins, extracellular matrix remodeling, and myocardial structural alterations [99, 100].

### 5.1. Paths by Which mtDNA Leaks out the Mitochondrial Matrix

Inhibition of abnormal opening of the mitochondrial permeability transition pore (mPTP) and enhancement of mitochondrial membrane stability can reduce the damage to cardiomyocytes induced by ROS [101]. mtDNA leakage processes, in addition to physical breakage caused by external forces, open some pathological pores or induce the transport of mtDNA into and out of the cell through carriers and mainly include the following four paths.

#### 5.1.1. mPTP Opening

The mPTP is a protein-based pore complex present at the contact point between the inner and outer mitochondrial membranes. When the mPTP opens, the mitochondrial transmembrane potential is lost, the mitochondria swell, the outer membrane ruptures, and mtDNA leaks into the cytoplasm. Studies have shown that strong inducers of mPTP opening include Ca^2+^ and ROS and that Ca^2+^ within the mitochondria is a key determinant of HF [102, 103]. It was found that blockage of mPTP opening and thus mtDNA leakage by inhibition of mitochondrial Ca^2+^ overload in myocardial I/R injury is beneficial [104].

#### 5.1.2. Bax and Bak Opening

Bcl-2-associated X protein (Bax) and Bcl-2 homologous antagonist killer (Bak) interact with components of the mPTP to regulate the opening and closing of the mPTP, which itself can form channels that affect apoptosis [97]. When the apoptotic pathway is activated, mtDNA is released into the cytoplasm [97, 105]. Knockdown of Bax provides protection in a mouse model of heart disease [106]. A recent study showed that DNA-dependent protein kinase catalytic subunits (DNA-PKcs) promote cardiac I/R injury by attenuating the ability of Bax inhibitor-1 (BI-1) to regulate mitochondrial homeostasis. When the function of BI-1 was restored, the ability of cardiomyocytes to resist myocardial I/R injury was also restored [107].

#### 5.1.3. GSDMD Pore Formation

When caspase-1 is activated, caspase-1 promotes the formation of pores in the plasma membrane by the pore-forming protein gasdermin D (GSDMD), leading to cellular scorching and mtDNA release [108]. A recent study found that GSDMD is required for enhanced early mobilization of neutrophils to the site of cardiac infarction and that knockdown of GSDMD in mice significantly reduces the infarct size, improves cardiac function, and increases survival after acute MI [109].

#### 5.1.4. Mitochondria-Derived Vesicle (MDV) Transport Pathway

**Figure 2:**
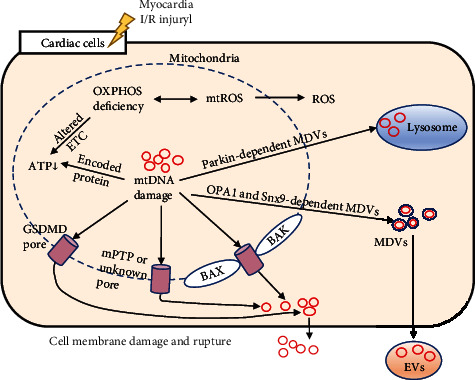
Pathways of mtDNA from mitochondria to the cytoplasm and extracellular matrix in cardiac cells. I/R: ischemia-reperfusion; OXPHOS: oxidative phosphorylation; ROS: reactive oxygen species; mtROS: mitochondrial ROS; ETC: electron transport chain; ATP: adenosine triphosphate; GSDMD: gasdermin D; mPTP: mitochondrial permeability transition pore; Bak: Bcl-2 homologous antagonist killer; Bax: Bcl-2-associated X protein; MDVs: mitochondria-derived vesicles; OPA1: optic atrophy 1; Snx9: sorting nexin 9; EVs: extracellular vesicles.

Mitochondria are capable of forming MDVs, and the formation of these vesicles increases with stress. MDVs transport substances between organelles and can swallow mtDNA [98]. In the circulation, MDVs carrying mtDNA can trigger inflammatory responses [110]. A recent study showed that MDVs can selectively regulate the transfer of mtDNA from damaged mitochondria within cells. On the one hand, the transport of mtDNA from damaged mitochondria to extracellular vesicles (EVs) requires the involvement of OPA1 and sorting nexin 9- (Snx9-) dependent MDVs [111]. These vesicles belong to a subset of MDVs, and previous studies have shown that they regulate the expression of mitochondrial antigens [112]. On the other hand, MDVs carrying mtDNA can be recognized and degraded by lysosomes through a process dependent on Parkin, a protein associated with Parkinson's disease, ensuring accurate translocation of damaged mitochondria to lysosomes for degradation and avoiding inflammatory responses caused by mtDNA-mediated activation of PRRs in the cytoplasm during translocation [111] (Figure 2).

### 5.2. Intracellular mtDNA and the Cardiac Inflammatory Response

**Figure 3:**
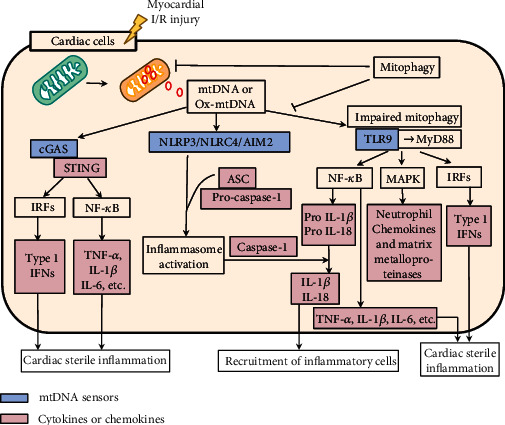
The released mtDNA activates mtDNA sensors in cardiac cells. mtDNA: mitochondrial DNA; Ox-mtDNA: oxidative mtDNA; cGAS: cyclic GMP-AMP (cGAMP) synthase; IFN: interferon; NF-*κ*B: nuclear factor-*κ*B; TNF-*α*: tnecrosis factor-alpha; IL-1*β*: interleukin-1beta; IL-6: interleukin-6; IL-18: interleukin-18; IRFs: IFN regulatory factors; TLR9: Toll-like receptor 9; MyD88: myeloid differentiation primary reactive protein 88; NLRP3: NLR family pyrin domain containing 3; NLRC4: NLR family CARD domain-containing protein 4; AIM2: absent in melanoma 2; MAPKs: mitogen-activated protein kinases.

Multiple PRRs encounter mtDNA and trigger a signaling cascade that activates the transcription factor NF-*κ*B and downstream signaling molecules, which in turn regulate target genes encoding proinflammatory cytokines and interferons in the heart [113] (Figure 3). Based on their subcellular localization, PRRs can be divided into two main categories. Intracellular PRRs include retinoic acid-inducible gene- (RIG-) I-like receptors (RLRs), nucleotide-binding and oligomerization domain- (NOD-) like receptors (NLRs), and absent in melanoma (AIM) 2 receptors. PRRs located on the cell membrane or intranuclear body include Toll-like receptors (TLRs) and c-type lectin receptors [114, 115].

#### 5.2.1. The mtDNA-TLR9 Pathway

In 2004, mtDNA was first discovered to induce an inflammatory response through its own unmethylated CpG motif [116], and TLR9 is the key receptor that recognizes the unmethylated CpG motif in mtDNA. Generally, TLR9 is localized on the endocytic membrane. mtDNA enters endocytic vesicles through the phagocytic process. mtDNA is recognized by TLR9 and stimulates the immune action of immune cells [117–119]. TLR9 is activated upon recognition of mtDNA, subsequently migrates to nuclear endosomes and lysosomes and activates mitogen-activated protein kinases (MAPKs) by binding to myeloid differentiation primary response protein 88 (MyD88) and NF-*κ*B, triggering an inflammatory response [119]. Oka et al. found that in DNase II knockout cardiomyocytes, mtDNA escapes autophagic degradation in the cytoplasm, leading to TLR9-mediated inflammatory responses, which in turn lead to the development of myocarditis and dilated cardiomyopathy in mice [35].

#### 5.2.2. mtDNA Activates the Inflammasome

The inflammasome, an important component of the inflammatory response, is a major source of interleukin-1*β* (IL-1*β*) and a promising new therapeutic target for CVD [120]. After myocardial ischemic injury, mtDNA and mitochondrial ROS are released into the cytoplasm, activating the NLR family pyrin domain containing 3 (NLRP3) inflammasome, triggering the assembly of cytoplasmic protein complexes in the inflammasome [121] and promoting inflammatory forms of cardiomyocyte death [122, 123]. The NLRP3 inflammasome is mainly expressed in macrophages, neutrophils, monocytes, and dendritic cells (DCs) [124, 125]. Furthermore, a recent study from our laboratory showed that hyperacetylation of mitochondrial proteins exacerbates inflammation by promoting the assembly of the NLRP3 inflammasome, which is closely associated with the severity of HFpEF [81]. Recently, adapters that inhibit the NLRP3 inflammasome and inhibitors that utilize NLRP3 inflammasomes were shown to be beneficial in reducing the myocardial infarct size and improving cardiac function in animal studies, and early clinical trials are expected to show their effectiveness [126]. A recent study found that the AIM2 inflammasome can also be activated by mtDNA [127]. Upon activation, AIM2 recruits precaspase-1, promoting caspase-1 activation and maturation of IL-1*β* and interleukin-18 (IL-18) [128, 129]. In addition, Jabir et al. found that mtDNA induces NLR family CARD domain-containing protein 4 (NLRC4) inflammasome activation [130]. Recently, the expression of the sensors of the four major inflammasomes (the NLRP1, NLRP3, NLRC4 and AIM2 inflammasomes) in patients with HF was assessed, and it was found that the expression of the inflammasome proteins AIM2 and NLRC4 was increased in patients with HF regardless of the etiology (ischemic or dilated cardiomyopathy), while the expression of NLR family pyrin domain containing 1 (NLRP1) and NLRP3 showed no change in HF samples [131]. Regarding inflammasomes, many related issues remain to be investigated. For example, it is unclear whether NLRP3 and NLRC4, which are receptor proteins, bind directly to mtDNA or whether other factors are involved.

#### 5.2.3. mtDNA Activates cGAS-STING

Studies have shown that mtDNA is a key factor in triggering the cyclic GMP-AMP (cGAMP) synthase- (cGAS-) STING pathway [132]. cGAS is mainly found in the nucleus [133]. cGAS senses cytoplasmic DNA and undergoes conformational changes to synthesize cGAMP, which binds to STING, causing STING activation and homodimerization and the recruitment of TANK-binding kinase 1 (TBK1), which phosphorylates interferon (IFN) regulatory factor (IRF) 3 and then phosphorylates the interferon gene [134]. In addition, STING activates the transcription factor NF-*κ*B, which synergistically induces the production of tumor necrosis factor- (TNF-) *α*, IL-1*β*, interleukin-6 (IL-6), and other inflammation-related factors through the TBK1-IRF3 pathway, triggering a proinflammatory response [135, 136]. In APP/PS1 mice with simulated Alzheimer's disease cardiac dysfunction, mice showed cognitive and myocardial defects, cardiac atrophy, cardiac interstitial fibrosis, reduced cardiomyocyte contraction, mitochondrial damage, and cytoplasmic mtDNA aggregation. In the presence of APP/PS1 mutations or melatonin administration, the significant decreases in cGAS and STING levels and the phosphorylation of TBK1 alleviated cytoplasmic mtDNA aggregation and improved cardiomyocyte appearance and function [137].

### 5.3. Extracellular mtDNA and the Cardiac Inflammatory Response

**Figure 4:**
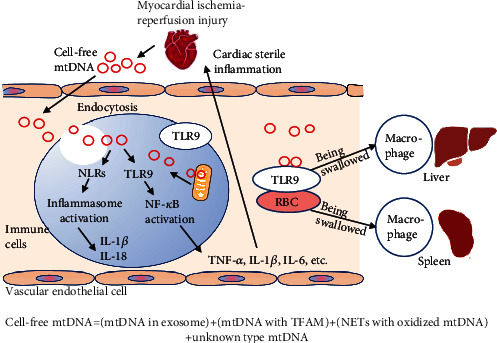
Inflammatory pathways activated by circulatory mtDNA. mtDNA: mitochondrial DNA; TFAM: transcription factor A; NETs: neutrophil extracellular traps; TLR9: Toll-like receptor 9; NF-*κ*B: nuclear factor-*κ*B; RBC: red blood cell; TNF-*α*: tumor necrosis factor-alpha; IL-1*β*: interleukin-1beta; IL-6: interleukin-6; IL-18: interleukin-18.

Mitochondria themselves and mtDNA in cardiomyocytes can be released into the extracellular space and transferred between cardiomyocytes [138]. Cell-free mtDNA can enter the circulatory system and travel naked or bind to TFAM in the circulation to form nucleoid structures [10], and both naked mtDNA and TFAM-bound mtDNA can be recognized by immune cells and initiate an innate immune response. mtDNA can also enter EVs [139] or be present in neutrophil extracellular traps (NETs) [140] (Figure 4). One study found that the only source of elevated plasma mtDNA levels after 40 min of cardiac I/R was the heart itself [141].

Studies on PRR activation in the circulation have focused on the TLR9 signaling pathway. Studies have reported that mtDNA release into the bloodstream after acute MI enhances myocardial I/R injury via the TLR9-P38 MAPK signaling pathway [142]. In another study, the hearts of WT mice were perfused with DNase I to inhibit the TLR9-mediated signaling pathway, and cardiac I/R was induced. The results showed that left ventricular systolic and end-diastolic pressure were improved and that the myocardial infarct size was reduced after reperfusion [143]. mtDNA also activates NLRs in circulating immune cells, triggering the assembly of inflammatory complexes in vesicles and the release of inflammatory factors and chemokines, leading to circulatory inflammatory responses [144]. Studies have shown that activation of the NLRP3 inflammasome increases susceptibility to sepsis in mice and that melatonin administration reduces mitochondrial damage and inflammation in sepsis [145]. The role of the cGAS-STING pathway in sepsis is controversial, as anaplastic lymphoma kinase (ALK) receptor expression is not detected in mononuclear macrophages in either humans or mice [146]. The cGAS-STING pathway is currently mainly reported to be involved in the formation of circulating mtDNA-induced NETs. It was found that in NETs, mtDNA-induced neutrophil extracellular trap formation (NETosis) is attenuated in STING-/- and TLR9-/- mice [147], but the relevance of the involvement of cGAS-STING in the formation of NETs to cardiac inflammation has not yet been reported. Notably, the turning point from systemic inflammation to myocardial inflammation is also unknown. Investigators believe that it occurs mainly through endothelial cell injury in the coronary arteries, which was described in more detail in a review by Mesquita et al. [148].

The extent to which mtDNA induces inflammatory responses in the circulation correlates with the extent of oxidative damage to mtDNA. For example, oxidative stress-induced elevation of extracellular mtDNA 8-oxyglucosyl levels increases the immunostimulatory capacity of mtDNA on plasmacytoid DCs (pDCs) [149, 150]. Scavenging of oxidized mtDNA is beneficial for reducing the systemic inflammatory response. Inside the cell, DNase within autophagic lysosomes plays a central role in mtDNA degradation [35]. However, no studies have confirmed whether deoxyribonucleases are also present in the circulation for digestion of cell-free mtDNA. Studies have shown that DNase administration after myocardial I/R injury reduces myocardial infarct size. However, the myocardial infarct area is larger after treatment with DNase and mtDNA inhibitors than after DNase treatment alone, which might be due to inhibition of the protective effect of nDNA in this pathway [151]. In addition, macrophages play an important role in the removal of mtDNA, and a recent study has shown that cardiomyocytes rely on exons to force damaged mitochondrial components into the extracellular space and that the extruded material is taken up and processed by macrophages residing in the heart through a network of phagocytic receptors called Mertks [152]. Circulating erythrocytes can bind to mtDNA via TLR9 [153]. A recent study found that DNA is recognized by TLR9 on erythrocytes and transported through the bloodstream to the liver and spleen, where it is taken up by macrophages. However, the binding of erythrocytes to DNA rich in CpG-based sequences also induces an inflammatory response [154].

## 6. Outlook

Early studies found higher circulating mtDNA concentrations in patients with acute MI with poorer prognosis than in patients with stable angina with better prognosis. Since then, an increasing number of studies have reported correlations between mtDNA levels in blood and the occurrence of clinical CVD [155]. In Table 1, we summarize the correlation between circulating mtDNA levels in humans and the occurrence of CVD, the need for cardiac surgery, the occurrence of clinical events or clinical indicators, including the predictive value of mtDNA for CVD prognosis, the ability of mtDNA to act as a marker of CVD, and the ability of mtDNA to mediate inflammation in CVD [156–169]. We found that the level of circulating mtDNA is elevated in most patients with CVD or during cardiovascular surgery. Similarly, in samples of myocardial tissue obtained during cardiovascular surgery, mtDNA levels were elevated [170], suggesting that circulating mtDNA is mainly derived from cardiac tissue that has been stressed and injured. Currently, the effect of mtDNA expression during extreme cardiovascular events, such as in cardiac arrest, on patient survival has not been determined [171].

A variety of studies on the treatment of CVD via restoration of normal mtDNA activity have been and are being conducted. Several preclinical studies have been successful in improving prognosis in CVD, particularly cardiac I/R injury. The successful strategies are described below.

(1) The early use of ischemic preconditioning mitigates oxidative stress in mitochondria and damage mtDNA. A recent study found that preconditioning has a similar protective effect on the heart in chronically obese patients. EVs containing oxidatively damaged mtDNA are released from adipocytes, enter the circulation, and are taken up by cardiomyocytes, inducing an antioxidant response in cardiomyocytes and thus protecting the myocardium from subsequent widespread acute oxidative stress damage [172]
(2) Restoration of the normal viability of mtDNA via improvements in the regulation of MLC and restoration of the mitochondrial quality control mechanism include mitochondrial fusion and fission balance, promotion of mitophagy [173], and facilitation of mitochondrial production [42–44]. A large number of antioxidants exert their antioxidant effects by enhancing mitochondrial function. Examples include resveratrol [174], polyphenols [175], coenzyme Q10 [176], and pioglitazone [177]. A recent study has shown that a large amount of mtDNA damage in mouse cardiomyocytes reduces NAD+ levels and that poly(ADP-ribose)polymerase (PARP) enzymes initiate mtDNA repair mechanisms requiring NAD+ [178]; thus, targeting NAD+ restoration has emerged as an attractive therapeutic modality for mtDNA repair and reversal of mitochondrial diseases [179–181]. In addition, a new mitochondrial quality control process, mitocytosis, in which damaged mtDNA is translocated to and subsequently removed from migrating vesicles upon exposure to mild mitochondrial stress, contributing to mediating intracellular mitochondrial homeostasis, was recently reported [182]
(3) Adjusting the ratio of WT mtDNA to mutant mtDNA increases the proportion of WT mtDNA in mitochondria. This includes the following:
  - The use of vectors for cardiac mitochondrial transplantation [183–186]: for example, EVs can facilitate the immediate transfer of mtDNA, and administration of mitochondria-rich EVs into the myocardium has been shown to have beneficial effects on in vivo cardiac function after MI [187]. A recent review article published by our laboratory details the therapeutic effects of nanodrugs and smart delivery systems in animal models of ischemic cardiomyopathy [188]
  - Isolation of mutated mitochondria and transfer of mitochondria to the extracellular compartment [189]
  - Generation of specific nucleic acid endonucleases to target and degrade mutated mtDNA [190]
  - Restoration of normal DNA via repair and editing of mutated mtDNA [191]
(4) Maintenance of mitochondrial membrane stability enhances the safety of mtDNA [107, 192, 193]
(5) Attenuation of cardiovascular injury is via inhibition of mtDNA and PRR-binding inflammatory pathways, mainly the TLR9 inflammatory pathway [194, 195], the NLRP3 inflammatory pathway [196–198] and the cGAS inflammatory pathway [199]
(6) There are cytoprotective effects of mitochondria-derived peptides encoded by mtDNA against CVD via maintenance of amitochondrial function and cell viability and alterations in nDNA expression during metabolic stress and cytotoxic injury [200]

In addition, it is also important to treat other diseases in the body, thus reducing their damage to the cardiovascular system. For example, the mtDNA level is elevated in the blood following acute ischemic kidney injury, and the heart shows altered oxidative stress and energy production [201]. Neuronal mtDNA damage in the nucleus of the solitary tract caused by neuroinflammation may be a central factor in the worsening of pressure reflex desensitization and subsequent cardiovascular dysfunction [202]. We hypothesize that rapid characterization and quantification of circulating mtDNA in real time will not only facilitate the diagnosis and treatment of CVD but will also be very beneficial for clinicians in determining the prognosis of multiple other comorbidities and minimizing progression to multiorgan failure.

## 7. Summary

In summary, an increasing number of studies have shown that CVD is closely related to mutations in mtDNA and to the inflammatory response induced by mtDNA, which acts as a DAMP. When mtDNA mutations accumulate to a point where the energy produced by cells falls below the minimum threshold of energy needed for tissues and organs to function, irreversible HF occurs. However, it is still not well understood how mutations in genes alter the structure and function of the proteins they encode and by what mechanisms these proteins with enzymatic functions affect mitochondrial OXPHOS and the production of energy from ATP. Moreover, in addition to mtDNA mutations, mutations in nDNA-encoded mitochondrial metabolism-related genes have been extensively linked to the development of CVD [13]. Impairment of mitochondrial function caused by environmental factors and lifestyle, among others, is also an important factor in the development of CVD.

Although early inflammatory responses are important for the clearance and repair of dead cells, chronic inflammation leads to long-term physiological remodeling of the heart [203]. Anti-inflammatory therapy for patients with CVD has not been shown to be effective for alleviating the mtDNA-induced innate immune response [204, 205]. Systemic inflammation- and comorbidity-driven microvascular dysfunction are increasingly recognized as drivers of cardiac remodeling in patients with HFpEF [206–209]. Recently, our laboratory generated a new mouse model of HFpEF that exhibits systemic inflammation, hypertension, obesity, and aging effects [81]. Several of our recent studies have also confirmed that the pathological mechanism underlying inflammation and HFpEF is driven by impaired mitochondrial metabolism [210–212]. This may be why anti-inflammatory regimens alone do not have a significant effect on slowing the course of CVD and suggest that improving mitochondrial dysfunction may be the most fundamental solution. As mtDNA is an important regulator of and participant in normal mitochondrial activity, reducing the proportion of mtDNA mutations and decreasing the inflammatory response to mtDNA should be an important avenue to combat CVD. In addition, a proper diet and effective exercise are also very effective interventions to improve cardiac mitochondrial metabolism. In conclusion, research on mtDNA is changing daily, and future in-depth studies will help to elucidate the mechanism of CVD from the perspective of mtDNA and provide new ideas for the prevention and treatment of CVD.

## Figures and Tables

**Table 1:** Correlation of circulating mtDNA measurements with clinical events or clinical indicators in humans with cardiovascular disease or cardiac surgery.

Circulating mtDNA function	Disease or operation	Study population	Experiment model	Response	Reference
As a prognostic marker for cardiovascular disease	Chronic HF	84 HF and 72 control participants	Plasma levels of mtDNA	Increased mortality in chronic HF patients with low mtDNA	PMID: 27349571
	ACS	11 patients with ACS survived and 3 patients died during 30 days after hospitalization	Peripheral blood of mt-cfDNA	Elevated mt-cfDNA increased mortality in ACS; the probability of death of patients with the increased level of blood plasma mtDNA (more than 4000 copies/ml) is 50%	PMID: 28049543
	Severe acute HF	90 severe acute HF and 109 consecutive chronic HF	Plasma levels of circulating mtDNA	Release of mtDNA with increased mortality in severe acute HF but not in chronic HF	PMID: 30632383
As a marker of cardiovascular disease	CHD	378 CHD and 378 healthy controls	mtDNA content of PBLs	A significant dose-response relation between increased CHD risk and lower mtDNA content	PMID: 25244506
	AMI	25 AMI, 25 with MI risk, and 20 healthy individuals	Plasma levels of mtDNA	mtDNA levels are elevated in AMI patients, but return to normal levels immediately after PCI	PMID: 25714070
	CHD with DM	50 CHD with DM2, 50 CHD patients without DM2, and 50 non-CHD-DM	Plasma ccf-mtDNA levels	ccf-mtDNA was elevated in type 2 diabetes with CHD and correlated with CRP levels	PMID: 26299063
	CHD with DM	50 CHD with DM2, 50 CHD patients without DM2 and 50 non-CHD-DM	Plasma ccf-mtDNA levels	ccf-mtDNA levels were elevated in CHD patients with DM compared with those without DM and non-CHD-DM	PMID: 26816608
	AF	54 AF and 104 non-AF controls	Peripheral blood of mt-cfDNA	AF was associated with an increased mt-cfDNA	PMID: 33737532
As a mediator of inflammation in cardiovascular disease	AMI	45 patients with AMI	Peripheral blood of mtDNA	High mtDNA with IL-1*β*, IL-6, IL-12p70, TNF-*α*, and TGF-*β*	PMID: 24797663
	CPB	38 patients undergoing CABG	Plasma mtDNA levels	High mtDNA with TNF-a, IL-6, and IL-8	PMID: 26104758
	CPB	68 patients undergoing CABG	plasma mtDNA levels	Higher mtDNA with TNF-a, IL-6 and higher activation levels of platelets	PMID: 27266529
	CPB	48 infants undergoing ventricular septal defect closure	Plasma mtDNA levels	High mtDNA with TNF-a, IL-6, and IL-8	PMID: 29174262
	AMI	38 patients with AMI and 33 control participants	Plasma mtDNA levels	Plasma mtDNA elevated after onset of AMI with higher WBC count, TNF-*α*, IL-6, and CRP	PMID: 27721319
	CPB	16 patients undergoing elective operations on CPB	Plasma levels of mtDNA	mtDNA was elevated following CPB; AF was seen in all patients with a ≥2-fold increase of mtDNA	PMID: 28487062

mtDNA: mitochondrial DNA; HF: heart failure; CHD: coronary heart disease; ACS: acute coronary syndrome. ccf-mtDNA: circulating cell-free mitochondrial deoxyribonucleic acid; DM2: type 2 diabetes; non-CHD-DM: patients without CHD and DM; CRP: C-reactive protein; PBLs: peripheral blood leukocytes; MI: myocardial infarction; AMI: acute MI; PCI: percutaneous coronary intervention; AF: atrial fibrillation; mt-cfDNA: mitochondrial-cell free DNA; CPB: cardiac surgery with cardiopulmonary bypass; CABG: coronary artery bypass graft.

## Data Availability

The data used to support the findings of this study are available from the corresponding author upon request.

## Abbreviations

AIM: Absent in melanoma
ALK: Anaplastic lymphoma kinase
AMPK: Adenosine 5′-monophosphate- (AMP-) activated protein kinase
ATP: Adenosine triphosphate
Bak: Bcl-2 homologous antagonist killer
Bax: Bcl-2-associated X protein
BI-1: Bax inhibitor-1
BNIP3 L: Bcl-2 interacting protein 3 like
cGAMP: Cyclic GMP–AMP
cGAS: Cyclic GMP–AMP (cGAMP) synthase
CoI: Cytochrome c oxidase I
CPC: Cardiac precursor cells
CVD: Cardiovascular diseases
CHD: Congenital heart disease
DAMPs: Damage-associated molecular patterns
DCs: Dendritic cells
D-loop: Displacement loop
DNA-PKcs: DNA-dependent protein kinase catalytic subunits
DNase: Deoxyribonuclease
Drp1: Dynamin-related protein 1
ETC: Electron transport chain
EVs: Extracellular vesicles
Fis1: Mitochondrial fission 1
FUNDC-1: FUN14 domain containing 1
GSDMD: Gasdermin D
HDAC: Histone deacetylase
HF: Heart failure
HFpEF: Heart failure with preserved ejection fraction
H-strand: Heavy strand
I/R: Ischemia-reperfusion
IFN: Interferon
IFN1: IFN type 1
LKB1: Liver kinase B1
IL-1: Interleukin-1
IL-18: Interleukin-18
IL-1*β*: Interleukin-1beta
IL-6: Interleukin-6
IRF: IFN regulatory factor
L-strand: Light strand
LONP1: Lon peptidase 1, mitochondrial
MAPKs: Mitogen-activated protein kinases
MDVs: Mitochondria-derived vesicles
MFF: Mitochondrial fission factor
Mfn1: Mitofusin 1
Mfn2: Mitofusin 2
MI: Myocardial infarction
Mid49: Mitochondrial dynamics proteins of 49 kDa
Mid51: Mitochondrial dynamics proteins of 51 kDa
mPTP: Mitochondrial permeability transition pore
mtDNA: Mitochondrial DNA
mtDNA-CN: mtDNA copy number
mtTFAM: Mitochondrial transcription factor A
MyD88: Myeloid differentiation primary response protein 88
ND: NADH dehydrogenase
nDNA: Nuclear DNA
NETosis: Neutrophil extracellular trap formation
NETs: Neutrophil extracellular traps
NF-*κ*B: Nuclear factor-*κ*B
NLR: NOD-like receptor
NLR: Nucleotide-binding and oligomerization domain (NOD)-like receptor
NLRC4: NLR family CARD domain-containing protein 4
NLRP1: NLR family pyrin domain containing 1
NLRP3: NLR family pyrin domain containing 3
NOD: Nucleotide binding and oligomeric domain
NRF1, NRF2: Nuclear respiratory factor 1 and 2
OPA1: Optic atrophy 1
OXPHOS: Oxidative phosphorylation;
PARP: Poly(ADP-ribose)polymerase
pDCs: Plasmacytoid dendritic cells
PGC-1*α*: Peroxisome proliferator-activated receptor *γ* coactivator 1*α*
PRR: Pattern recognition receptor
RIG: Retinoic acid-induced gene
RLR: RIG-I-like receptor
ROS: Reactive oxygen species;
SAHA: Suberoylanilide hydroxamic acid
Sfrp5: Secreted frizzled protein 5
SIRT1: Sirtuin 1
Snx9: Sorting nexin 9
STEMI: ST-segment elevation myocardial infarction
TBK1: TANK-binding kinase 1
TFAM: Transcription factor A
TLR: Toll-like receptor
TLR9: Toll-like receptor 9
TNF-*α*: Tumor necrosis factor-alpha
Wnt: Wingless-type
WT: Wild-type.
